# 5-Aminosalicylic acid for treatment of irritable bowel syndrome: A protocol for a systematic review and meta-analysis

**DOI:** 10.1097/MD.0000000000019351

**Published:** 2020-02-28

**Authors:** Wenyuan Cheng, Jing Li, Xiaoli Liu

**Affiliations:** a Department of Pharmacy, the First Hospital of Lanzhou University; b Department of Pharmacy, Gansu Gem Flower Hospital, Lanzhou, China.

**Keywords:** 5-ASA, IBS, irritable bowel syndrome, mesalazine, meta-analysis, protocol

## Abstract

**Background:**

The global prevalence of irritable bowel syndrome (IBS) is estimated to be as high as 15%, and it is estimated that IBS has a prevalence of approximately 10% to 20% in Western countries. Some trials showed mesalazine (5-aminosalicylic acid [5-ASA]) might be effective for IBS, but the results still need to be confirmed. Hence, this meta-analysis is designed to assess the efficacy and safety of mesalazine for IBS in adults and children.

**Methods:**

We conducted a comprehensive database search for randomized trials of mesalazine for IBS in PubMed, EMBASE, and the Cochrane Library. The search strategy was performed from inception to December 31, 2019, without restrictions on publication status and language. The reference lists of the included articles were also checked to identify additional studies for potential inclusion. Two reviewers will independently review all literature for inclusion and assess their risk of bias. Two reviewers will independently extract data from eligible studies based on a pre-designed standardized form. Any disagreements will be resolved by consensus. Stata SE 15.0 software will be used for data synthesis.

**Results:**

This is the first meta-analysis focusing on mesalazine for the treatment of IBS. We predict it will provide high-quality synthesis on existing evidence for IBS and a relatively comprehensive reference for clinical practice and development of clinical guidelines for IBS.

**Conclusion:**

This protocol outlined the significance and methodological details of a systematic review of mesalazine for IBS. This ongoing meta-analysis will provide high-quality synthesis on existing evidence for IBS.

**Registration:**

The meta-analysis has been prospectively registered in PROSPERO (CRD42019147860).

## 1 Introduction

Currently, the global prevalence of Irritable bowel syndrome (IBS) is estimated to be as high as 15%,^[1]^ and it is estimated that IBS has a prevalence of approximately 10% to 20% in Western countries.^[2]^ A combination of characteristic symptoms and the absence of warning signs on examination is used for IBS diagnosis, and the well-accepted Rome criteria now are in their fourth version.^[3]^ IBS is commonly classified into 4 main subtypes, that is, diarrhea-predominant (IBS-D), constipation-predominant (IBS-C), mixed (IBS-M), and unclassified (IBS-U). Moreover, IBS patients can also be divided into 2 categories, namely, nonspecific and post-infectious (PI-IBS).

Diverse factors, such as genes, psychosocial factors, brain-gut axis dysfunction, intestinal inflammation, intestinal microbiota alteration, as well as intestinal immune disruption, are all considered to play important roles in the pathogenesis of IBS.^[4,5]^ Currently, managements of IBS primarily aim at symptoms relief, that is, laxatives for constipation, antispasmodics for pain, anti-motility drugs for diarrhea, and antidepressants for mood and physical activity.^[5]^ The multiple and persistent symptoms of IBS contribute to high work absenteeism, high socioeconomic burden, and decline of life quality. IBS has been estimated to be the cause of between 8.5 and 21.6 days off work per year,^[6]^ and the chronicity of IBS symptoms leads to increased use of secondary health care services with health care costs of up to 4.1 billion Euros per year in Germany.^[7]^

Recent meta-analyses of randomized placebo-controlled clinical trials reported that mesalazine (5-aminosalicylic acid [5-ASA]) is the preferred first-line therapy for the acute treatment of mild-to-moderate ulcerative colitis.^[8]^ Mesalazine has a wide spectrum of pharmacological properties, but its exact mode of action is not yet clear.

It is reported that mesalazine may have an effect on activating peroxisome proliferator-activated receptors and promoting intestinal epithelial wound healing.^[9]^ Evidence from some clinical studies showed that the side effects of mesalazine were very low (5–10%), mild, and comparable to placebo,^[10]^ and the wide spectrum of biological activities of mesalazine is still increasing^[11,12]^; thus, mesalazine is expected to be effective, in addition to irritable bowel disease, also for IBS.

Evidence from clinical studies showed that low-grade intestinal inflammation plays a key role in the pathophysiology of IBS,^[13]^ and IBS patients exhibited significant increases of immune cells, macrophages, and enteroendocrine cells, in the lamina propria of colonic mucosa.^[14]^ This raises the possibility that intestinal anti-inflammatory agents might be effective in the treatment of IBS. It is reported that the pro-inflammatory mediators are of possible pathophysiological importance in IBS,^[15]^ and 1 study showed a reduction of intestinal mast cells and immune factors following treatment of mesalazine in 10 IBS patients.^[12]^ Moreover, an increasing number of reports have provided good evidence showing that there is an abnormal gut microbiota composition in IBS patients as compared with healthy controls,^[16]^ and mesalazine treatment reduced fecal bacteria abundance and rebalanced the major constituents of the microbiota.^[17]^

However, considering the potential risk to the above studies, the statistical power is small because of the small sample size, which might lead to misleading results. Therefore, the aim of this study is to include all available randomized trials of mesalazine for IBS and conduct a meta-analysis to assess the efficacy and safety of mesalazine for IBS in adults and children.

## 2 Methods

### 2.1 Patient and public involvement

There is no patient and public involvement in the whole process when we conduct this research.

### 2.2 Registration and reporting

The protocol of this systematic review and meta-analysis had been prospectively registered in PROSPERO (CRD42019147860) for quality control when we started searching for relative studies. Preferred Reporting Items for Systematic Review and MetaAnalysis (PRISMA)^[18]^ will be referenced throughout the study, and this protocol is based on an extension of PRISMA for protocol (PRISMA-P).^[19]^

### 2.3 Eligibility criteria

#### 2.3.1 Types of studies

Randomized controlled trials (RCTs) of mesalazine treatment for IBS will be considered for inclusion regardless of publication status and language of publication. Studies using active, no treatment, sham treatment, or placebo controls will be included. Trials with quasi-random designs will not be considered for inclusion.

#### 2.3.2 Participants

Adults and children with a diagnosis of IBS based on diagnostic criteria including Rome I, Rome II, Rome III, or Rome IV will be included. Appropriate participants will be included regardless of gender, race, educational status, or duration of IBS. Participants need to be able to participate in mesalazine treatment to be eligible for inclusion.

#### 2.3.3 Types of interventions

The types of interventions comprised trials that compared mesalazine as an oral formulation for the treatment of patients with IBS compared with placebo, or other formulations.

#### 2.3.4 Outcomes

Outcomes of interest in this study include: global or clinical improvement as defined by the included studies (e.g., IBS Severity Scoring System [IBS-SSS] or the Gastrointestinal Symptom Rating Scale [GSRS]); quality of life as measured by a validated quality-of-life scale (e.g., overall well-being, IBS Quality of Life Questionnaire [IBS-QoL], Short Form Health Survey [SF36]); adverse events; withdrawal due to adverse events; stool frequency; stool consistency (e.g., as rated by the Bristol Stool Scale); improvement in abdominal pain frequency and severity; depression; and anxiety.

However, various instruments are available to measure health-related outcomes in IBS,^[20]^ and the quality of these scales varies, and some may not be validated. This may be associated with bias; therefore, only the published and validated scales will be included in the full text process.

#### 2.3.5 Electronic searches

The following databases from inception to date will be searched to identify studies: the Cochrane Central Register of Controlled Trials (CENTRAL), MEDLINE, and EMBASE. The key text words of our search strategies are “IBS,” “irritable bowel syndrome,” “5-ASA,” “5-aminosalicylic acid,” and “mesalazine.” The search strategies will be customized for each database, and we will use recommended Cochrane search string for the identification of RCTs. All languages will be eligible for inclusion. References of included study will also be traced back to find potential qualified studies. Gray literature will be identified through Google Scholar.

#### 2.3.6 Study selection

**Figure 1:**
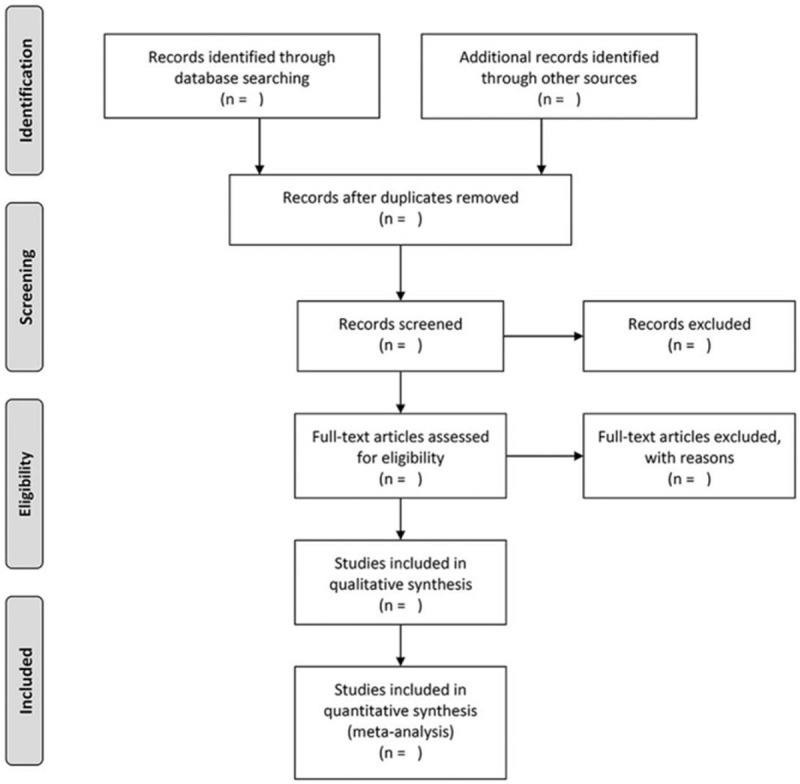
PRISMA flow diagram of studies selection process.

Literature records will be imported into Rayyan software for management after literature retrieval. We will exclude duplicates at first, and then 2 authors (CWY and LJ) will independently assess relevant abstracts and titles identified by the literature search against predefined inclusion criteria. A third author (XLL) will arbitrate any disagreement between authors. We will try to contact the corresponding authors if the full text cannot be obtained. The selection process will be presented in a PRISMA flow diagram (see Fig. 1).

#### 2.3.7 Data extraction

Two authors (CWY and LJ) will independently extract data from included studies. A third author (XLL) will arbitrate any disagreement between authors. If needed, the authors of the primary studies will be contacted for missing data and additional information. The Cochrane Handbook of Systematic Reviews of Interventions (5.2.0) will be referred to for guidance regarding any assumptions made about results.^[21]^ The characteristic information (e.g., author, year of publication, language, study setting, funding information, diagnostic criteria of participants, interventions, sample size, and duration of follow-up) and outcomes data (e.g., symptoms, quality of life, adverse events, stool frequency, stool consistency, depression, and anxiety) will be extracted too.

#### 2.3.8 Assessment of risk of bias in included studies

Quality components for each including RCT will be assessed for selection, detection, performance, reporting, and loss to follow-up bias. Factors to be assessed will include:

1. sequence generation,
2. allocation concealment,
3. blinding,
4. incomplete outcome data,
5. selective outcome reporting, and
6. other sources of bias.

Each item will be rated as high, low, or unclear risk of bias according to Cochrane Handbook of Systematic Reviews of Interventions (5.2.0),^[21]^ and justification from the study report will be supplied to support the judgment as appropriate. The studies will be reviewed independently and in duplicate (CWY and LJ). Disagreement will be resolved by discussion.

Grading of Recommendations Assessment, Development and Evaluation (GRADE) system will be used for rating overall quality of evidence supporting selected primary and secondary outcomes. In particular, randomized trials begin as high-quality evidence, but may be rated down by 1 or more of 5 categories of limitations:

1. risk of bias,
2. consistency,
3. directness,
4. imprecision, and
5. reporting bias.

The quality of evidence for each main outcome can be determined after considering each of these elements and categorized as either high, moderate, low, or very low.^[22]^ Reasons for downgrading the quality of the included studies will be reported in the footnotes of the “Summary of findings” table.

### 2.4 Data synthesis

For dichotomous outcomes, we will conduct a random effects meta-analysis using DerSimonian and Laird approach with risk ratios and report 95% confidence intervals. We will pool continuous outcomes with mean differences and 95% confidence intervals using DerSimonian and Laird random effects model. When continuous outcomes are deemed sufficiently similar but different scales have been used, the standardized mean difference (SMD) will be used to combine data. When standard errors instead of standard deviations (SD) are reported, we will convert the former to SD. We will not pool data for meta-analysis if a high degree of heterogeneity (*I*^2^ > 75%) is detected. We will assess statistical heterogeneity using Chi-Squared tests and I^2^ statistics. All primary analyses will be performed with STATA v15.1 (Stata Corp, College Station, TX).

### 2.5 Heterogeneity investigation

Cochrane Chi-Squared test and I^2^ will be used to quantitatively determine the heterogeneity (test level is a = 0.05). Significant heterogeneity is defined as *P* < .05. The magnitude of heterogeneity can be categorized as low (0%–30%), moderate (30%–50%), considerable (50%–70%), and substantial (70%–100%).^[21]^ To better interpret the source of heterogeneity, we will conduct exploratory subgroup analysis in addition to the above mentioned if applicable. If data are too heterogeneous to pool effect sizes in a meaningful or valid way, we will use a narrative approach to synthesize the data

### 2.6 Subgroups and sensitivity analysis

The following subgroups will be performed if the data are available: severity of IBS (e.g., constipation or diarrhea-predominant, severity of symptoms at baseline), different ages (adult versus adolescent), and gender (male versus female).

We will include a sensitivity analysis using either fixed-effect or random-effect models for meta-analysis depending on which was selected for primary analysis, as well as a sensitivity analysis to assess the potential influence of missing outcome data on our primary outcomes (both dichotomous and continuous). We will carry out a sensitivity analysis for the primary outcomes to explore how much variation between studies is explained by between-study differences in publication type, blinding, and studies at low risk of bias.

### 2.7 Ethics and dissemination

There is no need for a requirement of ethical approval and informed consent for this study because it is based on published literature. And the results of this systematic review will be submitted to a peer-reviewed journal for publication and information sharing.

## 3 Discussion

In recent years, randomized trials of mesalazine for IBS have been increasing continuously; however, it is still unsatisfactory in the diagnosis and therapy of the disease. The clinicians have not reached a consensus on the therapeutic principles and evaluations of IBS and lack normalized standards. At present, there are some clinical trials trying to find some effective and safe treatments for IBS; mesalazine is expected to be a potential option for IBS patients to improve the symptoms and health-related quality of life.

This meta-analysis will systematically evaluate the efficacy and safety of mesalazine for IBS patients by conducing meta-analysis of available randomized trials. And the results are hoped to provide a state of current research with precision results for clinical practice and future guideline development of IBS disease.

## Author contributions

WYC and XLL contributed to study concept and design, WYC and JL wrote the first draft, and all authors gave some suggestions for modification.
